# The epidemiology of ceftriaxone and cotrimoxazole-resistant *Escherichia coli* from humans, poultry, and their environment in central Malawi: A cross-sectional study

**DOI:** 10.1371/journal.pgph.0005869

**Published:** 2026-01-29

**Authors:** Ronald Chitatanga, Michael Luwe, Chikhulupiliro Yiwombe, Williams Mwantoma, Harry Milala, Catherine Kamwana, Stella Mazeri, Tiziana Lembo, Adrian Muwonge

**Affiliations:** 1 Antimicrobial Resistance National Coordinating Center, Public Health Institute of Malawi, Lilongwe, Malawi; 2 School of Biodiversity, One Health and Veterinary Medicine, University of Glasgow, Glasgow, United Kingdom; 3 Edinburgh Infectious Diseases, University of Edinburgh, Edinburgh, United Kingdom; 4 Fleming Fund Country Grant, University of North Carolina Project Malawi, Lilongwe, Malawi; 5 Mzuzu Agriculture Development Division, Ministry of Agriculture, Irrigation and Water Development (MoAIWD), Mzuzu, Malawi,; 6 Pharmacy department, Kamuzu Central Hospital, Lilongwe, Malawi; 7 Centeral Veterinary Laboratory, Ministry of Agriculture, Irrigation and Water Development (MoAIWD), Lilongwe, Malawi; North Carolina State University, UNITED STATES OF AMERICA

## Abstract

The poultry industry represents an important reservoir for clinical antibiotic resistance, especially in low- and middle-income-countries where food security pressures drive intensified farming and antibiotic use. However, evidence linking poultry production to public health risks remains limited in settings such as Malawi. This study examined *Escherichia coli* resistance to ceftriaxone and cotrimoxazole, two antibiotics with distinct stewardship priorities. We conducted a cross-sectional study between July and September 2022 in Malawi’s Central region, targeting poultry farms. Our mixed-methods design combined microbiological sampling from farm workers, poultry, and effluent water with a structured questionnaire on antibiotic use. *E. coli* isolates underwent antimicrobial susceptibility testing by disk diffusion for ceftriaxone and cotrimoxazole. 376 poultry farms were enrolled and among these, 17.6% reported using Trimovet, a veterinary formulation containing the same active ingredients as cotrimoxazole. Antibiotic use was more common among farms using commercial feed (aOR = 6.97; 95% CI: 3.43–14.17), and among farm workers with prior antibiotic knowledge (aOR = 5.56; 95% CI: 3.01–10.25). From 1,432 *E. coli* isolates recovered across all sources, resistance to cotrimoxazole was high: 80% in humans, 75% in environmental specimens, and 64% in poultry. In contrast, ceftriaxone resistance remained comparatively low (7.9%, 12%, and 7.3%, respectively). Predictors of cotrimoxazole resistance included farms in Nkhotakota district (aOR = 3.26; 95% CI: 1.84–6.01), fully housed chickens (aOR = 2.60; 95% CI: 1.67–4.08), commercial feed use (aOR = 1.54; 95% CI: 1.09–2.18), and prior antibiotic use (aOR = 1.67; 95% CI: 1.19–2.35). These findings highlight the risk that commercial poultry farming systems pose to public health, particularly for cotrimoxazole, which remains a cornerstone of Human Immunodeficiency Virus (HIV) prophylaxis and community care in Malawi. Strengthening antimicrobial stewardship across both human and animal sectors is urgently needed to mitigate transmission at the human–animal–environment interface.

## Introduction

Antimicrobial resistance (AMR) is an escalating global health challenge, which occurs when bacteria no longer respond to antimicrobials, rendering standard treatments ineffective and increasing the risk of severe illness, spread, and mortality [1]. In 2019, it was directly responsible for approximately 1.27 million deaths, with sub-Saharan Africa experiencing the highest mortality rate, 23.7 deaths per 100,000 of the population. Six bacterial pathogens were responsible for over 70% of this global burden, with *Escherichia coli* accounting for the largest share [1].

*E. coli*, classified by the World Health Organization (WHO) as a critical priority pathogen [2], has become increasingly resistant to key antibiotic classes, including Cephalosporins and Carbapenems [3]. This growing resistance complicates treatment of bloodstream and urinary tract infections in humans [1], and diarrheal diseases in livestock [4]. Addressing this challenge demands a One Health approach that integrates human, animal, and environmental health responses [5], thus, without such effective solutions, many countries may not meet their Sustainable Development Goal (SDG) targets, including those aimed at reducing poverty, improving health and supporting economic growth [5,6].

Both human and animal antibiotic use have accelerated the emergence and spread of antibiotic resistance (ABR) [7]. In the animal health sector, resistance has risen sharply. Between 2000 and 2018, global prevalence of resistance to tetracyclines, sulfonamides, and penicillin increased across chickens, pigs, and cattle [8]. In poultry, it rose from 15% to 41% [8]. Key pathogens, *E. coli*, *Campylobacter spp.*, *Salmonella spp.*, and *Staphylococcus aureus*, are now prioritized for surveillance due to their rising resistance profiles [8]. These trends threaten public health, food safety and food security, particularly in low-middle-income-countries (LMICs) [9].

In Malawi, systemic challenges such as limited regulation of antibiotic use in both human and animal sectors have compounded the AMR threat [10]. It can serve as an example of the challenges that many countries will likely face in the future. With a rapidly growing population (projected to double by 2042) and limited arable land, food security pressures will likely promote intensified animal farming [11], which may necessitate the adoption of unconventional practices, including extensive antibiotic usage. Malawian studies have already reported higher antibiotic use in intensive compared to extensive Malawian farms, particularly oxytetracycline, streptomycin, and erythromycin [10]. This highlights the potential contribution of livestock farming to the emergence and spread of resistant bacterial infections in the clinical setting.

The poultry sector is especially significant. It is one of the fastest-growing agricultural subsectors globally, expanding at 1.8% annually, and up to 2.4% in developing countries, a trend expected to continue through 2050 [12]. This predicted growth corresponds to an existing limited arable land (necessitating intensification) and the overuse of antibiotics which is likely to have a knock-on effect on the development of resistance [9]. Therefore, this explains why the Food and Agriculture Organization (FAO) highlights poultry systems as major contributors to AMR transmission to humans, particularly in settings where antibiotic use is poorly regulated [12].

**Fig 1:**
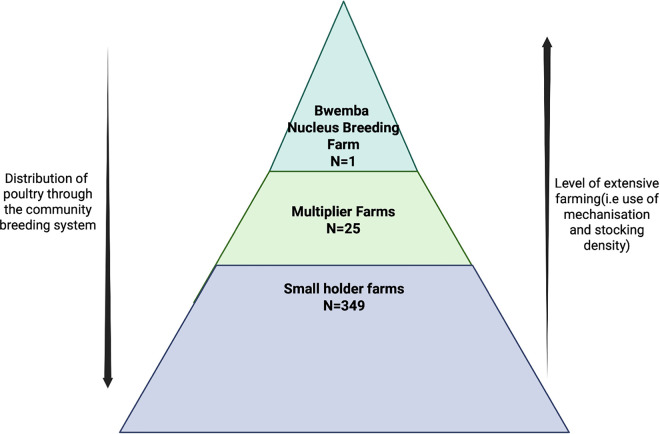
Poultry breeding system in the Central Malawian region. Bwemba Breeding Center, located in Lilongwe district, supplies the Central region with Black Australorp chickens. These chickens are supplied to small-holder farmers, through Moderate-scale farms.

In Malawi, the intensification of poultry production is expected to build upon a unique community-based breeding system established under the government-led Smallholder Poultry Improvement Programme (SPIP) [13]. Initiated in the 1950s, SPIP aimed to improve the productivity of indigenous chickens by crossbreeding them with Black Australorps, a breed shown to grow faster and yield more meat and eggs [13]. This locally adapted model continues to shape poultry production, particularly among smallholder farmers. The system operates as a three-tier structure (Fig 1), comprising government-run breeding centres (intensive commercial-scale operation) (e.g., Bwemba in Central Malawi), private multipliers (moderate-scale operation) who purchase improved breeds for redistribution, and smallholder farmers (family-scale operation) who typically rear the chickens under traditional management systems. This structure offers a valuable context to explore antibiotic use and AMR dynamics at the human–poultry–environment interface.

This study examined *E. coli* resistance to two antibiotics of contrasting stewardship priority: ceftriaxone, a third-generation cephalosporin in the WHO Watch category [14], and cotrimoxazole (trimethoprim–sulfamethoxazole), a widely used WHO Access category antibiotic recommended for prophylaxis in people living with the Human Immunodeficiency Virus (HIV) [15]. Investigating their resistance profiles not only provides insight into how stewardship strategies may shape resistance evolution, but also has direct public health relevance. Ceftriaxone is a critical treatment for severe infections in the Malawian health system, while cotrimoxazole remains a cornerstone of HIV care and infection prophylaxis. Resistance to these drugs in poultry-associated *E. coli* therefore raises concerns about transmission to humans through food, the environment, or direct contact, potentially undermining frontline treatments. By applying a One Health framework, this study aimed to: (1) identify farm-level factors associated with antibiotic use in poultry; and (2) estimate the proportion of *E. coli* isolates, recovered from various sources, that were resistant to ceftriaxone and cotrimoxazole; and (3) determine predictors of *E. coli* resistance across sectors. Ultimately, these findings illustrate how resistance emerging in animal production systems risks compromising human health by eroding the effectiveness of essential antibiotics and increasing the burden of difficult-to-treat infections.

## Methods

### Ethics statement

Ethical approval for this study was obtained from the National Health Sciences Research Committee (NHSRC) in Malawi (Protocol #22/05/2910). Additional approvals were granted by the Veterinary and Human Ethical Review Committees at the University of Edinburgh (VERC-78–21 & HERC-2021–732). Written informed consent was obtained from all participating farms who provided permission for study procedures to take place. Therefore, this study was conducted in compliance with all relevant ethical regulations.

### Study design and setting

**Fig 2:**
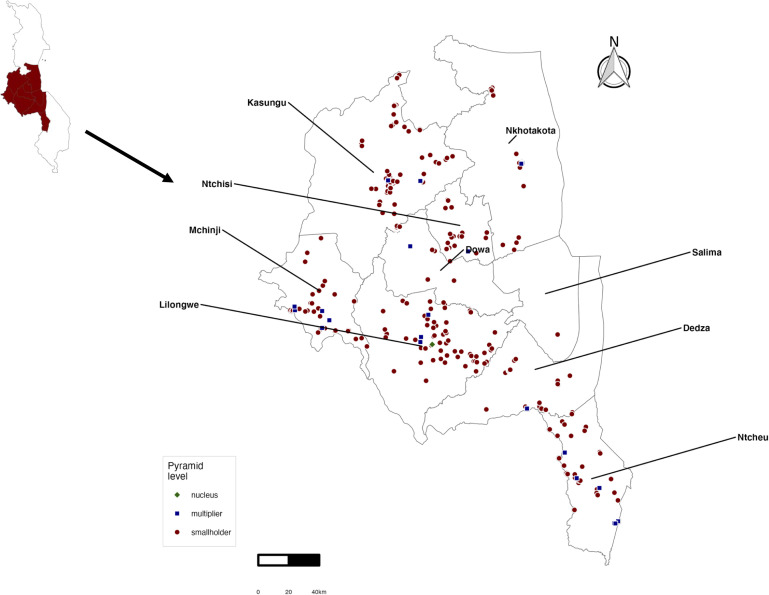
Spatial distribution of sampled poultry farms in Central Malawi. The intensive commercial-scale farm (Bwemba Breeding Center) is color-coded green, moderate-scale farms are blue, and family-scale farms red. Country boundary shapefile obtained from the GADM database of Global Administrative Areas (version 4.1; https://gadm.org), used under terms compatible with CC-BY 4.0. Map generated using R software.

This was a cross-sectional study which enrolled Black Australorp chicken farms from the Central region of Malawi. Malawi spans 118,480 km² and has 28 districts grouped into three regions: Northern, Central and Southern regions [11]. Data were collected from all nine districts in the Central region namely, Kasungu, Nkhotakota, Salima, Ntchisi, Dowa, Mchinji, Lilongwe, Dedza, and Ntcheu (Fig 2).

A mixed-method approach was adopted, which included collecting specimens from farmers (faecal specimens), chickens (cloacal swabs), and the farm environment (effluent water) for microbiological analysis as well as administering a structured questionnaire on antibiotic use. Data collection took place between 1 July 2022 and 30 September 2022.

### Study population

The study population comprised poultry farms registered in the Central region’s poultry database. Chickens in this region are supplied by the Bwemba Breeding Center (an intensive commercial-scale farm), and all farm categories in the distribution system were included, namely the breeding Center itself, moderate-scale farms, and family-scale farms.

### Eligibility criteria

The study included only healthy poultry older than six weeks. At the Bwemba Breeding Centre, eligible birds were of Black Australorp parent stock, while at moderate-scale farms, only healthy Black Australorps above six weeks of age were enrolled. On family-scale farms, both pure Black Australorps and their crossbreeds were eligible provided they were healthy and older than six weeks. Chickens showing signs of illness or younger than six weeks were excluded at all levels. Poultry farm workers were eligible if they were healthy (no gastrointestinal illnesses reported in the previous 14 days), aged 18 years or older, provided informed consent, and had direct stewardship responsibilities for the chickens. Farm workers were excluded if they had taken antibiotics within the previous 14 days, were younger than 18 years, or lacked direct poultry contact. Environmental sampling targeted effluent water sources within a 500-meter radius of each participating farm.

### Sampling frame

The Bwemba Breeding Centre was purposively selected due to its central role in distributing Black Australorp chickens. From 50 registered moderate-scale farms, 25 were randomly chosen. For family-scale farms, a sample of 349 was calculated using EpiTools (https://epitools.ausvet.com.au/oneproportion), assuming a total of 6,376 registered farmers, an expected prevalence of 60% based on a Nigerian study [16], and a 5% margin of error. Farm selection followed a random geographic sampling process using GPS mapping, with inaccessible sites excluded.

At the farm level, sample sizes (for farm workers, chickens, and environment samples) were determined independently. At Bwemba Breeding Center, 10 farm workers, 203 chickens, and one effluent water specimen were collected. The chickens sample size was based on a total flock size of 1,140, assuming an 80% resistance prevalence from a South African study [17] and a 5% margin of error. At moderate-scale farms, 25 farm workers, 242 chickens (10 per farm), and 25 environmental specimens were obtained. At family-scale farms, 345 farm workers, 690 chickens (2 per farm), and 345 environmental specimens were collected (one per farm).

### Data collection and processing

#### Generation of farm-level metadata.

At each farm, trained Research Assistants administered a semi-structured questionnaire via KoboToolbox [18] to capture farm-level metadata on demographics, poultry production, and antibiotic-related knowledge and practices. The tool included 41 questions across six domains, though only five (excluding knowledge of human antibiotics) were analysed for this study.

Demographic variables included age (18–29, 30–60, > 60 years), gender, education (primary, secondary, tertiary), poultry training (none, basic, advanced), and a unique Consent ID, which anonymised data and linked questionnaires to microbiological results.

Poultry production characteristics captured farm type (selling, consuming, both), flock size, chickens sold, production level (intensive commercial-scale, moderate-scale, family-scale), product type (meat, eggs, both), housing system (fully housed [birds kept indoors at all times], partially housed [birds housed indoors with controlled outdoor access for part of the day], free range [birds with regular unrestricted access to outdoor environments]), and commercial feed use (yes/no).

Antibiotic use data assessed knowledge and practices, including whether antibiotics were used, reasons for use, access pathways, visual identification of veterinary and human antibiotics, prescription-seeking behaviour, and duration of administration. These questions referred to general antibiotic use, not specific drugs. To elicit the visual recognition of antibiotics, Research Assistants employed visual aids (pictures of typical antibiotic packages and tablets found in Malawi.

#### Microbiological data.

Microbiological analyses were conducted at the National Microbiology Reference Laboratory (NMRL) in Lilongwe, Malawi. Cloacal swabs, human faecal samples, and environmental water samples were processed using standardized bacteriological methods, with full laboratory protocols provided in S1 Text.

Cloacal swabs were collected using liquid Stuart transport medium and, together with human faecal and water samples, transported under cold-chain conditions to the NMRL for processing within 24 hours. All specimens were cultured on MacConkey agar (with salts) and incubated for 18–24 hours at 35–37 °C in a 5% carbon dioxide atmosphere. Representative lactose-fermenting and non-lactose-fermenting colonies were selected and sub-cultured onto appropriate media to obtain pure isolates. Preliminary identification was performed using Gram staining, followed by biochemical testing. Identification of *Escherichia coli* was based on colony morphology, Gram-negative bacilli appearance, and biochemical reaction profiles consistent with established Enterobacteriaceae criteria as described by Lupindu [19].

Water samples underwent additional processing steps, including dilution in buffered diluent prior to culture, to account for the environmental matrix. Otherwise, identification procedures were consistent with those applied to cloacal and faecal samples.

Quality control measures were implemented throughout laboratory processing, including incubation of uninoculated media to monitor sterility, use of reference control strains (*E. coli* ATCC 25922), inclusion of blank and replicate samples for water testing, and confirmatory identification of a subset of isolates using the VITEK 2 automated diagnostic system [20].

Antimicrobial susceptibility testing was performed on confirmed *E. coli* isolates using the Kirby–Bauer disk diffusion method [21]. Although a broad antibiotic panel was tested, analyses in this study focused on ceftriaxone and cotrimoxazole resistance. Susceptibility interpretations were based on European Committee on Antimicrobial Susceptibility Testing (EUCAST) guidelines for *E. coli* [22]. Detailed laboratory procedures and antibiotic panels are described in S1 Text.

The final microbiology dataset consisted of four variables. These variables were, the Consent Identification number, the type of specimen (i.e., whether human, poultry or environmental) and the zone diameter recordings for ceftriaxone and cotrimoxazole.

### Statistical analysis

Data processing and analyses were performed in R (version 4.3.3) [23] following export from KoboToolbox and laboratory records. Farm metadata and microbiological datasets were merged by Consent ID using the *dplyr* package; records with missing identifiers or unmatched entries were excluded, resulting in a final dataset of 1,432 isolates. Data were cleaned and validated for completeness and consistency.

Descriptive statistics summarised farm and respondent characteristics, with categorical variables presented as frequencies and percentages, and continuous variables as medians with interquartile ranges (IQR). These are presented in the baseline characteristic table (Table 2).

**Table 2:** Respondent and farm production characteristics of 376 enrolled farms. The table summarises respondent characteristics and farm production characteristics of poultry farms. Proportions have been expressed as a percentage of the total of the category.

Characteristic	N = 376^1^
**Respondent characteristics**	
**Gender status**	
Female	182 (48%)
Male	194 (52%)
**Age range**	
18-29	41 (11%)
30-60	298 (79%)
> 60	37 (9.8%)
**Highest education attained**	
Primary	124 (33%)
Secondary	158 (42%)
Tertiary	94 (25%)
**Poultry training attained**	
None	236 (63%)
Basic	129 (34%)
Advanced	11 (2.9%)
**Farm production characteristics**	
**Poultry production level**	
Intensive commercial-scale	6 (1.6%)
Moderate-scale	25 (6.6%)
Family-scale	345 (92%)
**Farm type**	
Consuming	131 (35%)
Selling	11 (2.9%)
Both	232 (62%)
Unknown	2
**Farm product**	
Meat	16 (4.3%)
Egg	5 (1.3%)
Both	353 (94%)
Unknown	2
**Chicken sales in past year**	540 (148, 1,800)
Unknown	345
**Housing of chickens**	
Free range	200 (53%)
Fully housed	54 (14%)
Partially housed	122 (32%)
**Commercial feed use**	173 (46%)

^1^n (%); Median (IQR).

To explore predictors of farm-level antibiotic use, a generalised linear mixed-effects model (GLMM) was fitted with district as a random effect (Table 1). Frequencies and proportions of *E. coli* resistance to ceftriaxone and cotrimoxazole were calculated across human, poultry, and environmental sources, with differences assessed using Chi-square tests. Predictors of *E. coli* resistance were evaluated using two generalised linear models (GLMs) (Table 1).

**Table 1:** Characteristics of statistical models used in the analysis, showing the type of model, fixed and random effects.

Objective assessed by model	Type of Model	Main (fixed) effects	Random effects
**Objective 1**: Factors associated with antibiotic use on poultry (a farm using any antibiotic “yes” or not “no”)	Binomial GLMM	age range (18–29/30–60/60), gender (M/F), education (Primary/Secondary/Tertiary), poultry training status (none/basic/advanced), farm type (Selling/Consuming/Both), product (Meat/eggs/both), poultry production level (intensive commercial-scale/moderate-scale/family-scale), housing(fully housed/ free range/ partially housed), and prior knowledge about antibiotics (yes/no)	District (Dedza/ Dowa/ Kasungu/ Lilongwe/ Mchinji/ Nkhotakota/ Ntchisi/ Ntcheu/ Salima
**Objective 3A**: Factors associated with phenotypic *E. coli* resistance to ceftriaxone (an isolate being resistant to ceftriaxone “1” or susceptible “0”)	GLM	sample type (human/chicken/ environment), age-range, gender, education, poultry training status, farm type, product, poultry production level, housing, prior knowledge about antibiotics, prior knowledge about antibiotic resistance (Yes/no) and antibiotic use on poultry (Yes/no)	
**Objective 3B**: Factors Associated with phenotypic *E. coli* resistance to cotrimoxazole (an isolate being resistant to cotrimoxazole “1” or susceptible “0”)	GLM	Variables as above	

GLMM = Generalised Linear Mixed-effects Model.

Model refinement was performed via backward selection (*drop1()* function). Results are reported as adjusted odds ratios (ORs) with 95% confidence intervals (CIs). Assumptions and model fit were assessed using the *performance* package, with significance set at p < 0.05.

The full statistical workflow, including variables considered and interpretation steps, is provided in S1 Text. This work adhered to Strengthening the Reporting of Observational studies in Epidemiology (STROBE) guidelines for cross-sectional studies [24].

## Results

### Baseline farm and poultry production characteristics

A total of 376 farms were enrolled (Table 2). There were slightly more male farm workers (194/376, 52%) compared to female ones (182/376, 48%). Age-wise, many participants (298/376, 79%) fell within the 30–60-year range, with fewer younger (18–29 years old) participants (41/376, 11%). In terms of educational attainment, the majority (158/376, 42.0%) of respondents completed secondary education. Those with only primary-level education constituted 33% (124/376) of respondents, while 25% (94/376) attained tertiary-level education. Regarding professional poultry training, many farm workers (236/376, 63%) received no poultry training, while 34% (129/376) received basic training. A small fraction (11/376, 2.9%) of farm workers underwent advanced training.

The poultry production level varied among the 376 farms, with the majority (345/376, 92%) identified as family-scale farms (small holder). Moderate-scale farms constituted 6.6% (25/376) of the farms recruited. Many farms (232/376, 62%) practiced both consumption and selling of their product, where 94% (353/376) of the farms produced both meat and eggs. Regarding chicken sales over the past year, sales figures ranged widely, with a median monthly sales number of 540 (IQR 148 – 1800) chickens. However, sales data for 345 farms were not available due to poor record keeping.

Housing conditions for chickens also showed diversity. 53% (200/376) of the farms kept chickens on an extensive production system, characterised by free ranging, while 32% (122/376) were reared semi-intensively, characterised by a partial housing arrangement (where chickens were allowed outdoor access for part of the day). Only 14% (54/376) of farmers reared their chickens intensively, by keeping them fully housed everyday. Commercial feed usage was reported by 46% of the farms (173/376).

### Farm-level factors influencing antibiotic use

#### Visual-aid assessment of the knowledge of and use of veterinary and human antibiotics.

**Fig 3:**
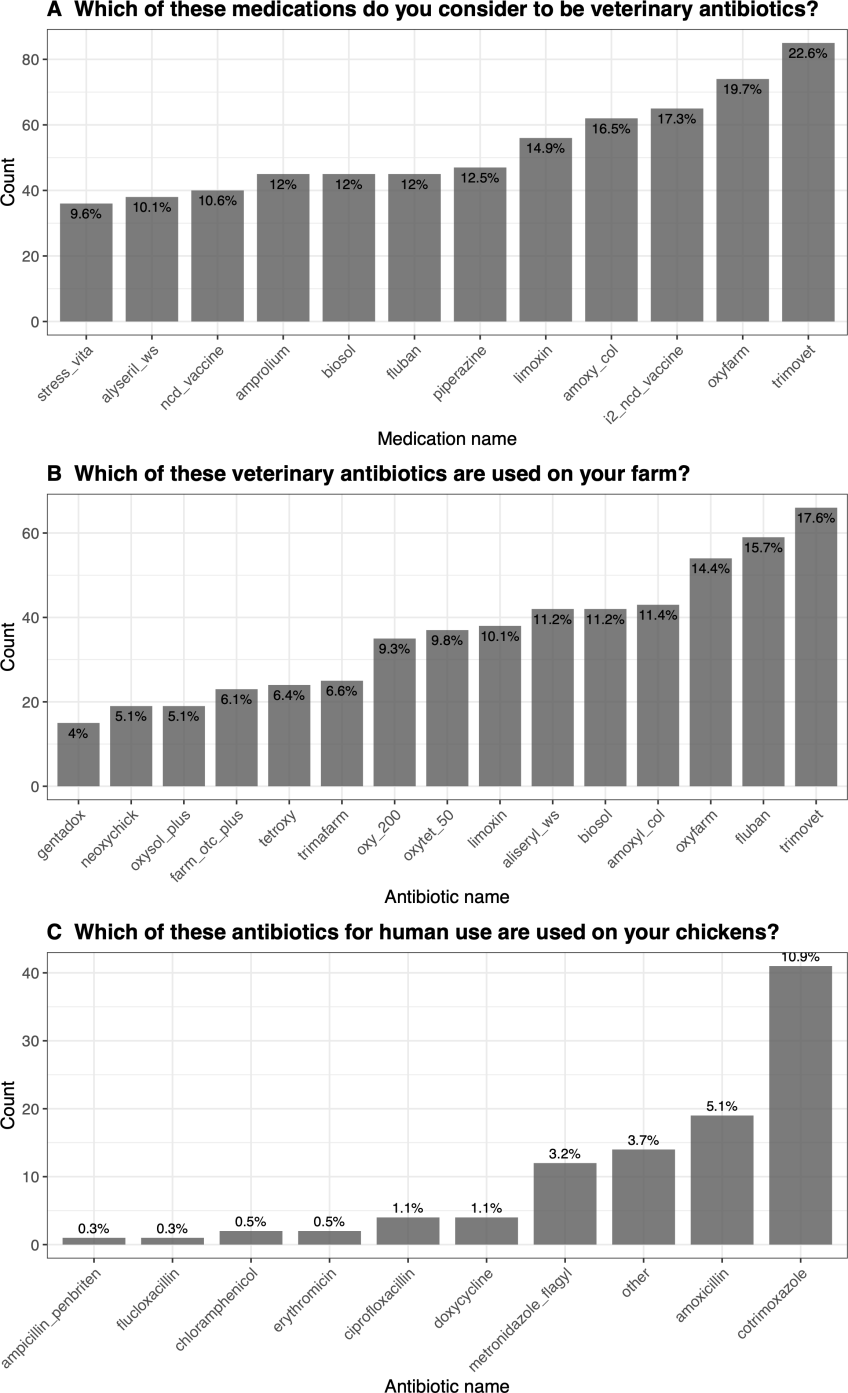
Farm workers’ identification, use of veterinary antibiotics and human antibiotics on poultry. This figure outlines the identification of veterinary antibiotics, their reported use and use of human antibiotics on farms. **A)** Outlines answers to a question qualifying their knowledge of antibiotics. **B)** Outlines answers characterising their use of veterinary antibiotics. **C)** Outlines answers characterising the cross-over use of human antibiotics on animals.

In response to the question “Which of these medications do you consider to be veterinary antibiotics?” (Fig 3A), Trimovet was most frequently reported as a veterinary antibiotic, by 23% (85/ 376 farms), followed by Oxyfarm (74/376, 20%) and, wrongly, i2 Newcastle Disease (NCD) vaccine (65/376, 17%). Other medicines like Stress Vita, Alyseril WS, and NCD vaccine were less frequently identified as veterinary antibiotics, by 9.6% (36/ 376), 10% (38/), and 11% (40/376) of farms, respectively.

In addition, responding to “Which of these veterinary antibiotics are used in your farm?” (Fig 3B), Trimovet was the most reported antibiotic in use, with 17.6% (66/376) of farms indicating its use, followed by Fluban 16% (59/376) and Oxyfarm 14% (54/376). On the lower end, Gentadox was the least reported, with only 4% (15/376) of farms reporting its use on their farms.

Finally, in response to “Which of these antibiotics for human use are used on your chickens?” (Fig 3C), cotrimoxazole and amoxicillin were identified as the most used drugs, with 10.9% (41/376) of enrolled farms using cotrimoxazole and 5.1% (19/376) using amoxicillin on their chickens.

### Predictors of antibiotic use on poultry

The GLMM of farm antibiotic use revealed significant associations between antibiotic use and several predictors (Table 3), after adjusting for random effects due to variability among districts. 22% of the variability in antibiotic use was attributable to differences between districts. The model explained 57% of the variance in antibiotic use based solely on the fixed effects (Marginal R² = 0.574) and 67% when considering both the fixed and random effects (Conditional R² = 0.669).

**Table 3:** Analysis of factors influencing antibiotic use in Central Malawi. The is a model output of a Generalized linear mixed-effects model evaluating the variability accounted by districts and fixed effects predictors.

		Antibiotic use on farm		
*Predictors*	*Category*	*Adjusted Odds Ratios*	*CI*	*p*
Gender	Female	–	–	–
	Male	0.48	0.27 - 0.87	**0.016**
Number of chickens		1.02	1.01 - 1.04	**0.011**
Commercial feed	No	–	–	–
	Yes	6.97	3.43 - 14.17	**<0.001**
Heard of antibiotics	No	–	–	–
	Yes	5.56	3.01 - 10.25	**<0.001**
**Random Effects**				
σ²	3.29			
^τ^00 _district_	0.95			
ICC	0.22			
N _district_	9			
Observations	376			
Marginal R^2^/ Conditional R^2^	0.574/ 0.669			

Regarding the predictors of antibiotic use, commercial feed use was a significant predictor. Specifically, the use of commercial feed was associated with a 6.97 times increase in the odds of using antibiotics (aOR 6.97, 95% CI: 3.43 – 14.17, p < 0.001) compared to those that did not use commercial feed. Additionally, prior knowledge of antibiotics was significantly associated with their use, where farmers who had heard of antibiotics were 5.56 times more likely to use antibiotics compared to those without such awareness (aOR 5.56, 95% CI: 3.01 – 10.25, p < 0.001). Furthermore, a small but significant increase in the odds of antibiotic use was associated with an increasing number of chickens, where for each additional chicken a farm kept, there was a 2% increase in the odds of using antibiotics (aOR: 1.02, 95% CI: 1.01 – 1.04, p = 0.011). Conversely, the odds of using antibiotics were significantly lower on farms operated by males compared to those operated by female farm workers, with 52% lower odds of using antibiotics (aOR 0.48, 95% CI: 0.27 – 0.87, p = 0.016).

Notably, other fixed effects evaluated during the backward model selection process were not statistically significant predictors of antibiotic use and were therefore excluded from the final model. These were age-range (p = 0.46), education of the farm workers (p = 0.46) and chicken housing status (p = 0.18). Additionally, poultry training, poultry production level, farm type, product and the number of chickens sold were not included in the final model due to a model failure to converge resulting from missing values.

### Proportion of phenotypically resistant *E. coli* isolates to ceftriaxone and cotrimoxazole

Of the 1,432 *E. coli* isolates recovered, 889 (62%) were from poultry, 331 (23%) from humans, and 212 (15%) from the environment (Table 4).

**Table 4:** *E. coli* isolate susceptibility to ceftriaxone and cotrimoxazole across chicken, environmental and human sources. The table summarises the percentages of isolates susceptible and resistant to each antibiotic, within each source.

Characteristic	Isolates from poultry samples, N = 889^1^	Isolates from environmental samples, N = 212^1^	Isolates from human samples, N = 331^1^	p-value^2^
**Susceptibility to ceftriaxone**				0.10
Susceptible	824 (93%)	187 (88%)	305 (92%)	
Resistant	65 (7.3%)	25 (12%)	26 (7.9%)	
**Susceptibility to cotrimoxazole**				<0.001
Susceptible	321 (36%)	53 (25%)	67 (20%)	
Resistant	568 (64%)	159 (75%)	264 (80%)	

^1^n (%).

^2^Pearson’s Chi-squared test.

**Fig 4:**
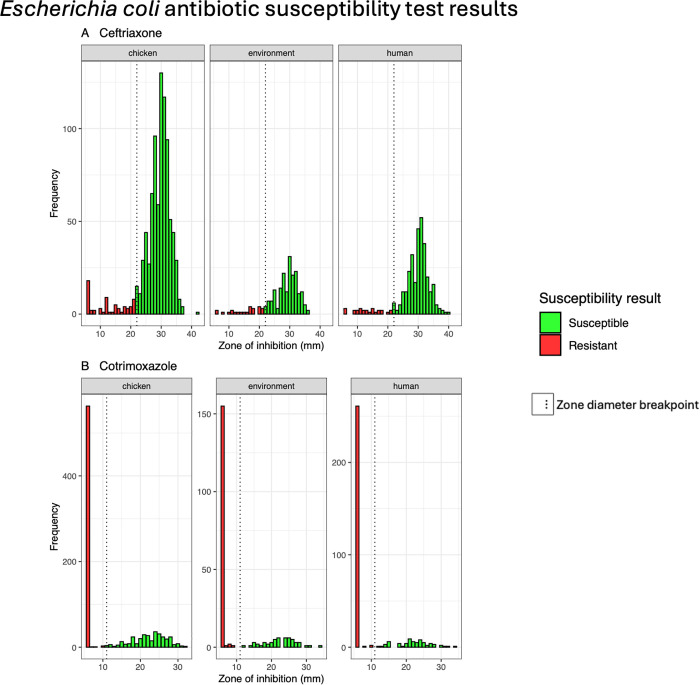
Antimicrobial susceptibility testing for *E. coli* in isolates retrieved from chicken, environment and human carriers. The diagram highlights the distribution of zone diameters recorded for each isolate (n = 1432) with susceptible isolates (green) falling above the breakpoint while resistant isolates (red) falling below this point. **A)** More *E. coli* isolates were susceptible to ceftriaxone than were resistant. This pattern was uniform in all sources. **B)** cotrimoxazole had more resistant *E. coli* isolates than susceptible ones and this was consistent across all sources.

For ceftriaxone, susceptibility was generally high across all sources (Fig 4A). Poultry-derived isolates showed the highest susceptibility at 93% (824/889), followed by human-derived isolates at 92% (305/331), and environmental isolates at 88% (187/212). Corresponding resistance rates were low: 7.3% in poultry, 7.9% in humans, and 12% in environmental isolates. However, these differences in resistance proportions were not statistically significant (*p* = 0.10).

In contrast, cotrimoxazole resistance was markedly higher and varied significantly by source (Table 4 and Fig 4B). Human isolates had the highest resistance rate at 80% (264/331), followed by environmental (75%, 159/212) and poultry isolates (64%, 568/889). These differences in resistance proportions were statistically significant (*p* < 0.001).

### Factors influencing *E. coli* resistance

#### Resistance to ceftriaxone.

The GLM explained 9.7% of the variance in ceftriaxone resistance based solely on the fixed effects (R^2^ Tjur = 0.097). Various significant predictors for resistance to ceftriaxone were identified (Table 5). The type of sample from which *E. coli* was isolated (i.e., whether from human, animal or environmental sources) was highlighted as a significant predictor. Environmental samples showed notably higher odds of containing ceftriaxone-resistant *E. coli*, with odds 2.99 (95% CI; 1.68 - 5.31, p < 0.001) times greater than poultry samples. Similarly, human-derived samples had increased odds of 1.75 (95% CI: 1.00 - 3.05, p = 0.046) times.

**Table 5:** GLM of factors influencing *E. coli* resistance to ceftriaxone in human, poultry and environmental sources. This model accounted for 9.7% of the variance.

		*E. coli resistance to ceftriaxone*		
*Predictor*	*Category*	*Adjusted Odds Ratios*	*CI*	*p*
Sample type	Poultry	–	–	–
	Environment	2.99	1.68 - 5.31	**< 0.001**
	Human	1.75	1.00 - 3.05	**0.046**
Production scale (pyramid strata)	Intensive commercial scale	–	–	–
	Moderate-scale	0.05	0.01 – 0.14	**< 0.001**
	Family-scale	0.24	0.11 - 0.50	**< 0.001**
Farm type	Consuming	–	–	–
	Selling	1.33	0.57- 3.12	0.510
	Both (Consuming & selling)	0.54	0.32 - 0.93	**0.023**
Commercial feed	No	–	–	–
	Yes	1.63	0.96 – 2.79	0.073
Antibiotics use	No	–	–	–
	Yes	1.73	0.94 – 3.36	0.087
Observations		1410		
R^2^ Tjur		0.097		

Additionally, the scale of production (whether Intensive commercial scale or moderate-scale or family-scale) influenced the carriage of *E. coli* resistant to ceftriaxone. Specifically, moderate-scale farms demonstrated a 95% (aOR 0.05, 95% CI: 0.01 – 0.14, p < 0.001) reduction in odds, and family-scale farms demonstrated a 76% (aOR 0.24, 95% CI: 0.11-0.50, p < 0.001) reduction, when compared to the intensive commercial-scale farm.

Furthermore, the influence on *E. coli* resistance to ceftriaxone using commercial feed and antibiotics on the farm was also examined. However, these factors did not play a significant role with reported odds ratios of 1.63 and 1.73 for commercial feed use and antibiotic use, respectively.

#### Resistance to cotrimoxazole.

The GLM on *E. coli* resistance to cotrimoxazole explained 11.9% of the variance based solely on the fixed effects (R^2^ Tjur = 0.119). Identified significant predictors (Table 6) included the sample type from which *E. coli* was isolated, where human-derived samples showed higher odds of containing cotrimoxazole-resistant *E. coli*, with 3 (95% CI: 2.19 – 4.15, p < 0.001) times increased odds than the poultry samples. Similarly, environmental samples had increased odds of 2.10 (95% CI: 1.48 – 3.03, p < 0.001) times.

**Table 6:** GLM of factors influencing *E. coli* resistance to cotrimoxazole in human, animal and environmental sources.

		*E.coli* resistance to cotrimoxazole		
*Predictors*	*Category*	*Adjusted Odds Ratios*	*CI*	*p*
Sample type	Poultry	–	–	–
	Environment	2.10	1.48 – 3.03	**<0.001**
	Human	3.00	2.19 – 4.15	**<0.001**
District	Lilongwe	–	–	–
	Dedza	1.16	0.71 – 1.89	0.552
	Dowa/Salima	1.10	0.50 – 2.62	0.823
	Kasungu	0.93	0.64 – 1.36	0.706
	Mchinji	1.16	0.74 – 1.84	0.517
	Ntchisi	0.89	0.56 – 1.40	0.604
	Ntcheu	0.76	0.50 – 1.15	0.196
	Nkhotakota	3.26	1.84 – 6.01	**<0.001**
Housing	Free range	–	–	–
	Fully housed	2.60	1.67 – 4.08	**<0.001**
	Partially housed	0.72	0.52 – 1.01	0.055
Commercial feed	No	–	–	–
	Yes	1.54	1.09 – 2.18	**0.015**
Heard about antibiotics	No	–	–	–
	Yes	0.63	0.45 – 0.87	**0.006**
Use antibiotics	No	–	–	–
	Yes	1.67	1.19 – 2.35	**0.003**
Awareness about resistance	No	–	–	–
	Yes	1.46	1.07 – 1.99	**0.018**
Observations		1432		
R^2^ Tjur		0.119		

Additionally, when compared to samples from Lilongwe district, *E. coli* specimens originating from Nkhotakota showed a 3.26 (95% CI: 1.84 – 6.01, p < 0.001) times increase in the odds of resistance to cotrimoxazole. No other district showed a statistically significant result. Furthermore, at the farm level, chickens that were fully housed, compared to free range chickens, had 2.60 times (95% CI: 1.67 – 4.08, p < 0.001) increased odds of for carrying cotrimoxazole-resistant *E. coli*.

Chicken feeding practices also showed notable contribution to cotrimoxazole resistant *E. coli* carriage. The model showed 1.54 times increased odds (aOR 1.54 [95% CI: 1.09 – 2.18, p = 0.015]) on farms that used commercial feed compared to those farms not using commercial feed.

Finally, the GLM identified farmers prior knowledge of antibiotics, antibiotic use and existing knowledge about antibiotic resistance as significant predictors for the carriage of cotrimoxazole resistant *E.coli.* Farmers with prior knowledge of antibiotics had 37% (aOR 0.63 [95% CI: 0.45 – 0.87, p = 0.006]) reduced odds compared to those with no prior knowledge of antibiotics. Farms that used antibiotics, when compared to those that did not, had a 1.67 (95% CI: 1.19 – 2.35, p = 0.003) times increase in odds of carrying cotrimoxazole resistant *E. coli*. Prior awareness of antibiotic resistance increased the odds of carrying cotrimoxazole-resistant *E. coli* by 1.46 times (aOR 1.46 [95% CI: 1.07 – 1.99, p = 0.018]) when compared to no awareness about antibiotic resistance.

### Model comparison of predictors of antibiotic use and resistance

**Fig 5:**
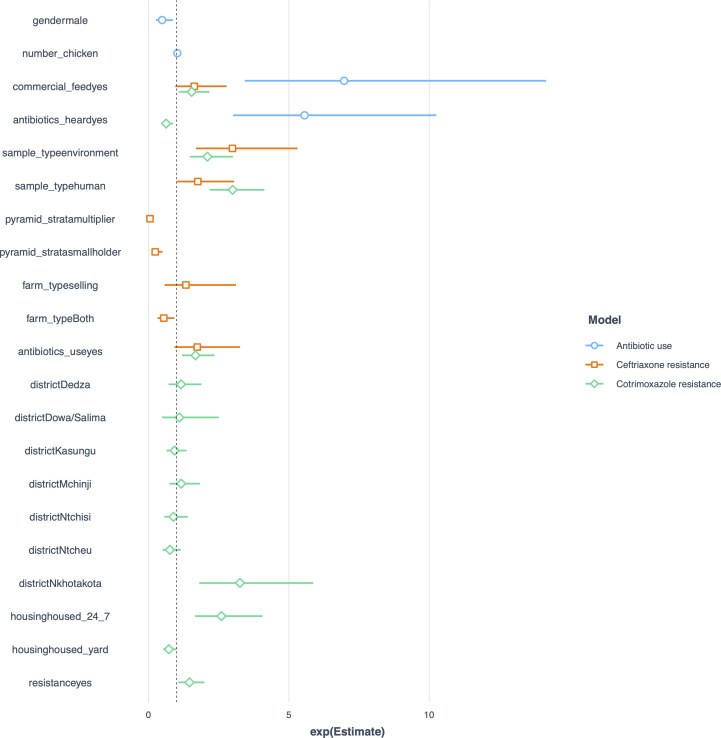
Adjusted odds ratios from the three logistic regression models. This forest plot displays the adjusted odds ratios (with 95% confidence intervals) from three models assessing predictors of: (1) reported antibiotic use in poultry farms, (2) ceftriaxone-resistant *Escherichia coli*, and (3) cotrimoxazole-resistant *E. coli*. Values greater than 1 indicate increased odds of the outcome, while values below 1 indicate decreased odds.

Three logistic regression models were developed to examine the predictors of (1) reported antibiotic use, (2) ceftriaxone-resistant *Escherichia coli*, and (3) cotrimoxazole-resistant *E. coli*. Across all models (Fig 5), commercial feed use emerged as the only significant predictor increasing the odds of antibiotic use and *E. coli* resistance to either antibiotic. In the resistance models, environmental and human sample types were more likely than poultry samples to yield resistant *E. coli*. Additionally, antibiotic use in the farm increased the likelihood of *E. coli* resistance to either drug.

## Discussion

This study revealed that the most used antibiotic among poultry farmers in central Malawi was Trimovet, a veterinary formulation of sulphamethoxazole and trimethoprim, with additional evidence of cross-over use of cotrimoxazole intended for humans. Antibiotic use was more likely on farms with larger flocks, those using commercial feed, those managed by women, or where farm workers already had prior knowledge of antibiotics. Alarmingly, *E. coli* resistance to cotrimoxazole was widespread across human, poultry, and environmental samples. This pattern highlights the potential for resistance amplification across interconnected reservoirs in a human-to-environment-to-animal transmission pathway, rather than a unidirectional spillover from poultry to humans. While ceftriaxone resistance was comparatively lower, its detection in human and environmental isolates is concerning, given its critical role in treating severe infections in hospitals. Notably, resistance to both antibiotics was more prevalent in human and environmental isolates than in poultry, underscoring the spillover risk to other exposed populations. Farms located in Nkhotakota district, with permanent poultry housing, prior antibiotic use, and reliance on commercial feed, showed particularly high odds of harbouring cotrimoxazole-resistant *E. coli*. Collectively, these findings reinforce how antibiotic practices in food production may shape resistance patterns relevant to human health, while recognising that causal relationships cannot be inferred from this cross-sectional study.

To our knowledge, this is the first cross-sectional study in Malawi to integrate human, animal, and environmental health data to characterize antimicrobial resistance patterns and explain antibiotic use behaviours. These findings underscore the importance of a One Health approach in AMR surveillance and provide an evidence base for developing targeted antimicrobial stewardship interventions.

Cotrimoxazole is both widely accessible and frequently used in Malawi’s health system. As a WHO “Access” category antibiotic [14], it is recommended for prophylaxis against bacterial infections in HIV-infected patients in sub-Saharan Africa [25]. The high rates of *E. coli* resistance to cotrimoxazole observed in our study likely reflect this extensive and long-standing use within Malawi’s healthcare system, with spillover into environmental and animal reservoirs. Importantly, cotrimoxazole resistance in commensal *E. coli* has been shown to be highly prevalent across LMICs and is recognised as reflecting long-term, population-level antibiotic pressure rather than recent antimicrobial exposure [26]. Our findings are consistent with this literature: while pooled estimates suggest a prevalence of approximately 67% cotrimoxazole resistance in commensal *E. coli* across LMICs [26], we observed an even higher prevalence among human isolates in our study (80%), underscoring the entrenched nature of cotrimoxazole resistance in this setting. Clinical data from a recent multi-centre analysis of over 2,700 isolates collected from seven hospitals across Malawi (2020–2024) reinforces this pattern, reporting alarmingly high resistance to cotrimoxazole among Gram-negative organisms (72.7–89.7%) [27]. This alignment between clinical findings and our community-level observations underscores the pervasive nature of cotrimoxazole resistance in Malawi, reinforcing the urgency of addressing its widespread and cross-sectoral use through strengthened stewardship, regulation, and surveillance efforts.

This pattern of use may be worsened by weak enforcement of prescription-only drug regulations, which permits widespread unsupervised access. For example, in Zambia, Malawi’s neighbour, nearly 100% of surveyed pharmacies were noted to dispense antibiotics without a prescription according to a 2023 narrative review [28]. Malawi faces similar regulatory challenges, contributing to the unsupervised over-the-counter access to cotrimoxazole. In fact, Mankhomwa, Tolhurst [10] reported the cross-over use of cotrimoxazole in food-producing animals in southern Malawi in a cross-sectional study, a trend further quantified by our study where 10.9% of enrolled farms have reported this practice. The widespread availability of cotrimoxazole in the community underscores the urgent need for strengthened but appropriate regulatory and monitoring systems targeting prescription only drugs to counteract this concerning ABR trend.

Intensified farming techniques have been consistently associated with higher levels of antibiotic use, a trend supported globally by literature, indicating how antibiotics are often employed prophylactically in these settings to meet the economic demands of improved productivity and biosecurity [29,30]. In contrast, extensive farms tend to use antibiotics primarily for therapeutic purposes, resulting in comparatively lower usage [29,30]. A 2022 Malawian cross-sectional study focusing on cattle, sheep and poultry farms also found higher antibiotic utilisation in intensive farms compared to extensive ones [10], highlighting that farms in low-income settings are similarly affected by this trend. These findings underscore the importance of monitoring and controlling antibiotic utilisation on intensive farms in resource-restricted settings. Given that these intensive farms are typically larger in size and more likely to use commercial feeding approaches, it is unsurprising that our study aligned with this contribution by intensive farms.

In addition to the role played by intensified farming practices, our findings revealed that prior awareness of antibiotics among farm operators was significantly associated with their use, underscoring how knowledge and perception shape antibiotic-related decisions. Thus, a critical need for targeted educational interventions that promote behavioral change, especially within intensive farming systems where misuse is more likely. Evidence from high-income settings supports the value of such interventions, for instance, a systematic review of seven studies in the United States reported that educational programmes led to reductions in antibiotic use by cattle farmers of up to 37% [31]. However, while such programmes are increasingly being adapted to low-resource settings, our study brings attention to a key contextual consideration: gender dynamics in poultry health management. Specifically, we observed that women were more likely than men to use antibiotics on their flocks. Therefore, any educational intervention aiming to reduce antibiotic misuse must be gender-sensitive, incorporating these sociocultural dynamics to ensure relevance and effectiveness.

Conversely, the uniformly lower prevalence of *E. coli* resistance to ceftriaxone, compared to the resistance towards cotrimoxazole, observed in this study suggests that this antibiotic remains effective against *E. coli* strains circulating across human, poultry, and environmental domains in Malawi. A systematic review of studies published between 1990 and 2019 across sub-Saharan Africa reported a median prevalence of 18.4% (IQR: 10.5–35.2) for *E. coli* resistance to third-generation cephalosporins, including ceftriaxone, in bloodstream infections among hospitalized patients [32]. While direct comparisons should be made cautiously, given that the systematic review focused on clinically infected individuals, whereas this study assessed healthy carriers, the markedly lower prevalence of resistance (7.9%) in our community-based work highlights a critical window of opportunity. Specifically, it suggests that community reservoirs of *E. coli* may not yet reflect the elevated resistance observed in clinical settings, offering a chance to prevent further transmission and escalation. As ceftriaxone is classified as a WHO “Watch” category antibiotic and plays a central role in the empirical treatment of severe infections in Malawi’s health system, these findings underscore the urgent need to strengthen antimicrobial stewardship initiatives at both hospital and community levels to preserve its effectiveness.

Building on the need to preserve ceftriaxone’s clinical efficacy, our study explored contextual factors that may contribute to emerging resistance in non-clinical reservoirs. Although the overall prevalence of ceftriaxone-resistant *E. coli* was low, several farm-level predictors merit attention. Notably, isolates from human and environmental specimens were more likely to show resistance than those from poultry, suggesting that an anthropogenic pathway may play a larger role in ceftriaxone resistance than direct veterinary use. Ceftriaxone is broadly used in African hospitals, including Malawi, where its once-daily dosing and broad-spectrum activity make it a preferred first-line treatment in resource-constrained settings [33,34]. However, its high utilization may inadvertently introduce resistant strains into the environment, especially in the absence of hospital wastewater treatment (HWW) systems. A meta-analysis by Zhang, Huang [35] underscored the role of untreated HWW in disseminating resistance genes into surrounding communities. In Malawi’s Central region, all districts have at least one hospital prescribing Ceftriaxone, yet none are currently equipped with on-site HWW treatment facilities targeting drug residues. This raises concerns about environmental contamination with drug-resistant organisms, particularly around densely populated or poorly drained settings. These findings reinforce the necessity of integrating environmental health infrastructure, such as wastewater management, into AMR containment strategies, especially to prevent the downstream effects of clinical antibiotic use on community and environmental resistance patterns.

In the analysis that uncovered the determinants of cotrimoxazole resistant *E. coli*, intensified farming techniques were implicated as significant drivers. Specifically housing conditions and commercial feed use. Housing conditions for chickens, particularly continuous housing versus free ranging, have been shown to influence the risk of antibiotic resistance. Studies have suggested that continuously housed chickens, often reared in high-density environments, are more susceptible to infections due to overcrowding and other stress-related factors, leading to a higher reliance on prophylactic antibiotics [36,37]. In contrast, free ranging typically involves fewer flock sizes and expression of more natural behaviours, which reduces stress and the need for routine antibiotic use [36,37]. In our study, farms that permanently housed chickens demonstrated significantly higher odds of harbouring cotrimoxazole-resistant *E. coli*, aligning with this evidence and reinforcing the concern that intensive confinement may contribute to increased resistance pressures.

Although this study did not analyse the specific antibiotic content of commercial poultry feed, the widespread recognition of cotrimoxazole among farmers suggests its prominent role, either through direct administration or potential inclusion in feed. Similar patterns have been observed elsewhere; for instance, studies in Bangladesh detected antibiotics such as ampicillin, ciprofloxacin, and enrofloxacin in commercial poultry feed, which contributed to significant residue levels in broiler meat [38]. While cotrimoxazole was not specifically reported in that context, it is plausible that in other low-resource settings like Malawi, feed may contain subtherapeutic levels of sulfonamides as a cost-effective prophylactic measure. Such unregulated inclusion could drive the emergence of resistance even in the absence of documented usage. These findings underscore the need for regulatory oversight and quality control in feed manufacturing, alongside the promotion of better husbandry practices to mitigate AMR risks.

An anticipated finding in this study was the significant association between farm-level antibiotic use for poultry and the presence of AMR [7,39]. However, a more striking and unexpected observation was the role of geographic location, specifically in farms located in Nkhotakota district, which exhibited significantly higher odds of harbouring cotrimoxazole-resistant *E. coli* compared to Lilongwe, the reference district. This elevated resistance may be explained by undocumented contextual farming practices prevalent in Nkhotakota. Unlike urbanized Lilongwe, Nkhotakota is a rural district characterized by more informal and less-regulated farming systems which may have limited access to veterinary oversight. These conditions collectively increase the risk of unregulated antibiotic use. While causality cannot be conclusively inferred from our cross-sectional data, the district disparity highlights a crucial reservoir of resistance that merits focused epidemiological and stewardship attention.

Despite providing a comprehensive examination of the role of the poultry industry in the emergence of antibiotic resistance, this study presents several limitations that must be acknowledged. Firstly, the cross-sectional nature of the study restricts the ability to establish causality between the observed patterns of antibiotic usage and the development of resistance. This limitation complicates the understanding of whether increased resistance drives higher antibiotic usage or vice versa, which is crucial for designing effective interventions.

Another significant limitation arises from the data collection methods used, primarily the reliance on self-reported data for antibiotic use practices. This approach may lead to reporting biases where respondents either underreport or overreport their use of antibiotics due to recall issues, particularly in regions where antibiotic use might be regulated or stigmatised. Additionally, while the study employed standardised techniques for identifying *E. coli* and assessing resistance, the variability in environmental conditions and sample handling might introduce inconsistencies in the data, affecting the reliability of the findings. However, this risk was minimised using standard operating procedures for handling microbiological specimens.

Finally, the statistical approach employed in this study also introduces specific limitations. The use of a backward variable selection method in the predictive model development process may not always result in the most robust model. This method, while useful for reducing complexity, might overlook significant predictors that are only apparent when a forward selection approach is also applied. By not utilising a more robust method that combines both backward and forward selection, there is a potential risk of bias in the final model, which could affect the interpretation of the data regarding factors influencing antibiotic resistance. Additionally, this study did not include molecular analyses to identify specific genes associated with antimicrobial resistance, which limits the mechanistic understanding of the observed resistance patterns. Therefore, future research investigating the role of the poultry industry in ABR emergence in central Malawi should consider incorporating more robust predictive modelling techniques or developing a causal modelling approach, and molecular analysis to improve the accuracy and reliability of the findings.

## Conclusion

We highlight the multifactorial drivers of resistance, including intensified farming practices, gendered management roles, prior antibiotic awareness, and the use of commercial feed. Importantly, the observed geographical disparity points to the need for localized antimicrobial stewardship strategies. Furthermore, we underscore the value of a One Health approach in designing context-specific public health interventions that address both antibiotic use and resistance in community and agricultural settings. Moving forward, improved regulatory oversight, environmental safeguards, and gender-sensitive education initiatives will be key to mitigating the spread of antibiotic resistance in Malawi and similar low-resource settings.

## Supporting information

S1 Text Laboratory procedures and generation of microbiology data. Statistical analysis methods.
(DOCX)

S2 Text Farm-level questionnaire. (PDF)

S1 Checklist (PDF)

## Abbreviations

ABR: Antibiotic Resistance
AMR: Antimicrobial Resistance
AMS: Antimicrobial Stewardship
CI: Confidence Interval
E. coli: Escherichia coli
GLM: Generalized Linear Model
GLMM: Generalized Linear Mixed-Effects Model
HIV: Human Immunodeficiency Virus
HWW: Hospital Wastewater
ICC: Intraclass Correlation Coefficient
IQR: Interquartile Range
NCD: Newcastle Disease
NMRL: National Microbiology Reference Laboratory
OR: Odds Ratio
PPS: Point Prevalence Survey
R²: Coefficient of Determination
R² Tjur: Tjur’s Coefficient of Discrimination
WHO: World Health Organization
