# Supplementary material for: The epidemiology of ceftriaxone and cotrimoxazole-resistant Escherichia coli from humans, poultry, and their environment in central Malawi: A cross-sectional study
Source: PLOS Glob Public Health. 2026 Jan 29;6(1):e0005869. doi: 10.1371/journal.pgph.0005869 (PMC12854471; doi:10.1371/journal.pgph.0005869)
Supplement: S1 Text — Laboratory procedures and generation of microbiology data. Statistical analysis methods. (DOCX) [file pgph.0005869.s001.docx]

**ADDITIONAL FILE 1**

**Laboratory procedures and generation of microbiology data**

1. **Cloacal swab sample processing**

Cloacal samples were collected using liquid Stuart transport medium swabs and transported under cold-chain conditions to the National Microbiology Reference Laboratory (NMRL) within 24 hours of sample collection. Upon receipt, swabs were cultured directly onto MacConkey agar (with salts) and incubated for 18–24 hours at 35–37 °C in a 5% carbon dioxide atmosphere. Following incubation, plates were examined for bacterial growth, and representative lactose-fermenting and non-lactose-fermenting colonies were selected. Pure single colonies were sub-cultured onto MacConkey agar (with salts) and nutrient agar to obtain pure isolates and incubated for a further 18–24 hours under the same conditions. Pure colonies were subjected to Gram staining to confirm Gram-negative bacilli morphology. Biochemical identification was performed using a standard panel of tests including catalase, oxidase, indole, methyl red, urease, sulfide–indole–motility (SIM), triple sugar iron (TSI), and citrate utilisation tests. Selected biochemical tests (urease, SIM, TSI, and citrate) were incubated for an additional 18–24 hours prior to interpretation. Identification of *Escherichia coli* was based on characteristic colony morphology on MacConkey agar, Gram stain appearance, and biochemical reaction profiles consistent with established Enterobacteriaceae identification criteria outlined by Lupindu (1). These criteria included formation of pink colonies on MacConkey agar because of lactose fermentation, microscopic appearance of small single, rod-shaped (bacilli) bacteria that are Gram-negative (staining pink or red due to their thin peptidoglycan layer) and biochemical test results consistent with a positive indole reaction, a positive methyl red test and negative result for citrate utilization tests.

**2. Human faecal sample processing**

Human faecal samples were collected in sterile stool containers and transported to the NMRL within 24 hours of collection under cold-chain conditions. In the laboratory, samples were similarly cultured on MacConkey agar (with salts) and incubated for 18–24 hours at 35–37 °C in a 5% carbon dioxide atmosphere. After incubation, representative lactose-fermenting and non-lactose-fermenting colonies were sub-cultured onto MacConkey agar (with salts) and Blood Agar plates to obtain pure isolates, followed by incubation for an additional 18–24 hours. Pure colonies were Gram stained to confirm Gram-negative morphology and subjected to biochemical identification like the cloacal sample processing approach. Identification of *E. coli* was done in line with the process for cloacal samples.

**3. Water sample processing**

Environmental sampling involved the collection of water samples from wastewater effluent points associated with poultry housing units. For each sampling point, 10 mL of water effluent was aseptically collected into sterile Falcon tubes and transported under cold-chain conditions within 24 hours. In the laboratory, samples were diluted 1:10 using buffered diluent, and the final dilution was used for bacterial culture. A volume of 100 µL was spread onto MacConkey agar (with salts) and incubated for 18–24 hours at 35–37 °C in a 5% carbon dioxide atmosphere. Plates were examined for growth, and representative lactose-fermenting and non-lactose-fermenting colonies were sub-cultured onto MacConkey agar and Blood Agar plates to obtain pure isolates. Following incubation, pure colonies were Gram stained and subjected to biochemical testing like those done for cloacal samples and fecal samples. *E. coli* was identified once the above criteria was met.

**Specimen transport and storage**

To maintain specimen integrity during transportation, a cold-chain system was employed for all sample types. Samples were transported in cooler boxes containing ice packs and delivered to the National Microbiology Reference Laboratory in Lilongwe District for processing. Where immediate processing was not feasible, specimens were stored at 4–7 °C and processed within 24 hours.

**4. Quality control and confirmatory identification**

Quality control procedures were implemented throughout laboratory processing to ensure the accuracy and reliability of bacterial isolation and identification. To assess media sterility and exclude laboratory contamination, uninoculated MacConkey agar plates were incubated overnight at 37 °C alongside inoculated plates. The absence of microbial growth on uninoculated control plates confirmed that any bacterial growth observed on inoculated media originated from the study samples rather than environmental or laboratory contamination. In addition, reference control strains, including *Escherichia coli* ATCC 25922, were used as positive controls to validate culture characteristics, biochemical identification, and antimicrobial susceptibility testing procedures.

For environmental water samples, additional quality control measures were applied. Blank samples were processed and incubated in parallel with true samples to verify that sampling equipment and handling procedures were free from contamination. Sample replicates were also included to assess reproducibility and detect potential variability in bacterial recovery. Any discrepancies observed between replicate samples were investigated to identify possible procedural or environmental sources of variation.

To further validate organism identification, a subset of *E. coli* isolates confirmed through conventional culture, Gram staining, and biochemical testing were randomly selected and subjected to confirmatory identification using the VITEK 2 automated diagnostic system, a proven automated bacterial identification and susceptibility testing system that uses fluorescence-based technology (2).

**Antimicrobial susceptibility testing (AST)**

Once *E. coli* was identified, AST was performed using the disk diffusion (Kirby-Bauer) method (3). The AST included fourteen antibiotics: ampicillin (10μg), cefotaxime (5μg), ceftazidime (10μg), ceftriaxone (30μg), chloramphenicol (30μg), ciprofloxacin (5μg), ertapenem (10μg), imipenem (10μg), meropenem (10μg), gentamicin (10μg), nitrofurantoin (100μg), piperacillin (30μg), tigecycline (15μg) and cotrimoxazole (25μg). For the data analysis in this study, only AST data related to ceftriaxone and cotrimoxazole was used. Breakpoints were assessed according to the European Committee on Antimicrobial Susceptibility Testing (EUCAST) guidelines for *E. coli* using the two antibiotics (4). The breakpoint for ceftriaxone resistance was 22mm and that for cotrimoxazole was 11mm. All *E. coli* isolates below these breakpoints were considered resistant and those above were considered susceptible to the antibiotic tested.

**ADDITIONAL FILE 2**

**Statistical analysis**

***Descriptive Statistics***

Initially, descriptive statistics were made for the enrolled farms. Frequencies and percentages were computed for demographic characteristics containing categorical variables (i.e. age range, gender, education level and poultry training attendance). For numerical variables (i.e. number of chickens in the farm and number of chickens sold), median and interquartile range (IQR) were computed to capture central tendency and dispersion, respectively.

***Objective 1: Farm- level factors associated with antibiotic use on poultry***

Initially, in every farm, all enrolled farm attendants underwent a drug bag assessment- where they were presented with a visual-aid of medications to evaluate their knowledge of antibiotics, use of veterinary antibiotics on the farm and any use of human antibiotics on poultry. This information was summarized as a frequency plot (**Figure 4**).

In addition, to investigate the factors associated with antibiotic use on poultry, a generalised linear mixed-effects model (GLMM) was developed (**Table 1**). In this model, the response variable was antibiotic use, which was treated as a binary variable where “yes” pertained to farms that used antibiotics and “no” to those that did not. The null hypothesis for this model suggested no significant association between farm-level characteristics and the likelihood of using antibiotics. This model was fitted using a binomial distribution, with a logit link function.

Predictive modelling, rather than causative modelling, was employed in this analysis. The significance of all farm-level metadata was evaluated using a backward selection process. The `drop1()` function was used to initiate this process. Initially, a GLMM including all variables (age range, gender, education, poultry training status, farm type, produce, poultry production level, housing, and prior knowledge about antibiotics) was developed. Insignificant predictors were subsequently removed based on their impact on improving model prediction. The district where the farm was located, with nine levels, was included as a random effect. The final model output was expressed as odds ratios with 95% confidence intervals (CIs) to outline the impact of these factors on the likelihood of antibiotic use on the farm.

***Objective 2: Proportion of E. coli isolates phenotypically resistant to ceftriaxone and cotrimoxazole***

To investigate the proportion of *E. coli* isolates, retrieved from poultry, human and environmental sources, with phenotypic resistance to ceftriaxone and cotrimoxazole, frequencies and percentages of resistant and susceptible isolates were computed. In addition, Chi square tests were calculated to evaluate whether there were any statistical differences between proportions of resistant and susceptible isolates retrieved from human, poultry and environmental carriers.

***Objective 3: farm-level factors associated with phenotypic E. coli resistance to ceftriaxone and cotrimoxazole***

To examine factors associated with phenotypic *E. coli* resistance to ceftriaxone and cotrimoxazole, two generalized linear models (GLMs) were developed, one for each antibiotic (**Table 1**). The outcome variables were binary, coded as "1" for resistant and "0" for susceptible isolates, based on zone diameter readings interpreted according to EUCAST breakpoints. Each model was fitted using a binomial distribution with a logit link function. The null hypothesis for these models posited no significant association between farm-level characteristics and the likelihood of carrying a resistant *E. coli* isolate to either ceftriaxone or cotrimoxazole.

Similarly, predictive modelling was employed in this analysis. All explanatory variables (from farm-level metadata) were included in the initial GLM. The explanatory variables included sample type, age-range, gender, education, poultry training status, farm type, produce, poultry production level, housing, prior knowledge about antibiotics, prior knowledge about antibiotic resistance and antibiotic use on the farm. Subsequently, insignificant predictors were removed based on their impact on improving model prediction, using the *drop1()* package. The final model output was expressed as odds ratios with 95% confidence intervals. All these steps were similar in the GLMs predicting *E. coli* resistance to ceftriaxone and cotrimoxazole and with similar initial predictors tested.

**References**

1. Lupindu AM. Isolation and Characterization of Escherichia coli from Animals, Humans, and Environment. InTech; 2017.

2. Guido Funke DM, Chiara deBernardis, Alexander von Graevenitz, Jean Freney. Evaluation of the VITEK 2 System for Rapid Identification of Medically Relevant Gram-Negative Rods. Journal of Clinical Microbiology 1998;36(7).

3. Hudzicki J. Kirby-Bauer Disk Diffusion Susceptibility Test Protocol. American Society for Microbiology. 2009.

4. E. Matuschek DFJB, G. Kahlmeter. Development of the EUCAST disk diffusion antimicrobial susceptibility testing method and its implementation in routine microbiology laboratories. Clinical Microbiology and Infection. 2013;20(4).
