# Supplementary material for: The epidemiology of ceftriaxone and cotrimoxazole-resistant Escherichia coli from humans, poultry, and their environment in central Malawi: A cross-sectional study
Source: PLOS Glob Public Health. 2026 Jan 29;6(1):e0005869. doi: 10.1371/journal.pgph.0005869 (PMC12854471; doi:10.1371/journal.pgph.0005869)
Supplement: S2 Text — (PDF) [file pgph.0005869.s002.pdf]

Fleming_Malawi_Questionnaire

Remember to obtain a SIGNED consent form and add a BARCODE on it

Select fellow

- ( ) Catherine
- ( ) Chikhulupiliro
- ( ) Harry
- ( ) Mike
- ( ) Ron
- ( ) Williams

Select RA name conductinng survey

Have you obtained consent?

- (x) yes
- ( ) no

Thank you for your time

Questions for people who consent

Scan barcode from the consent form

Did scan fail (if yes manually enter below)

- ( ) yes
- ( ) no

» If barcode fails

Manually enter sample code (first time)

Manually enter sample code (second)

Record farm level in the poultry pyramid

- ( ) nucleus
- ( ) multiplier
- ( ) smallholder

District

- Lilongwe
- Dedza
- Ntchisi
- Dowa
- Kasungu
- Nkhotakota
- Ntcheu
- Mchinji
- Salima

Record the gps location

latitude (x.y °)

longitude (x.y °)

altitude (m)

accuracy (m)

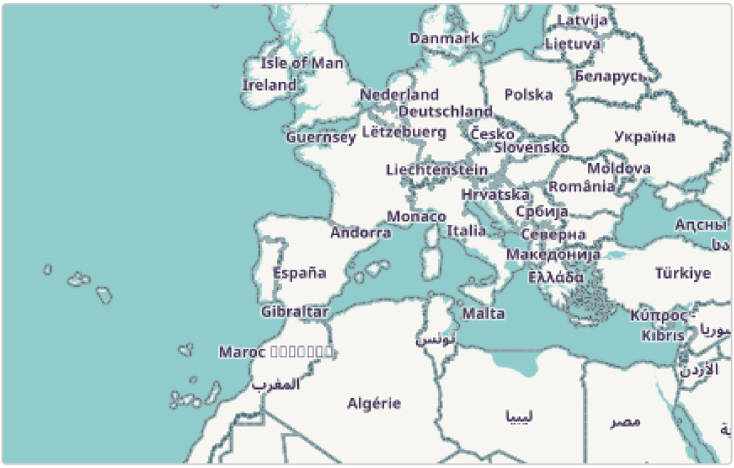

Did gps fail?

- yes
- no

If gps fails, record manually (Longitude)

If gps fails, record manually (Latitude)

Is this the person who provided a human sample?

- yes
- no

Record gender

- male
- female

Which age category are you?

read out age categories

- 18 to <30
- 30 to 60
- >60

What is your highest educational qualification?
maphunziro anu munafika nawo pati?

- ◯ no formal education
- ◯ primary school
- ◯ secondary school
- ◯ higher level qualification

Have you received any poultry production training?
Kodi munachitapo maphunziro a kawetedwe ka nkhuku?

- ◯ none (no training at all)
- ◯ basic (short training in poultry keeping)
- ◯ advanced (formal training certificate or above)

How many chickens do you have (smallholder farmer?)
Muli ndi nkhuku zingati?

____

How many chickens do you have as parent stock (multiplication or nucleus farm)?
Muli ndi makolo a nkhuku angati? (kuchulutsa kapena pa phata la famu)

____

How many chickens have you sold in the last 12 months (multiplication or nucleus farm)?
Mwagulitsako nkhuku zingati muchaka chapitachi?

____

» Districts where chickens are bought

Which district do you get your replacement Australorp chickens or eggs from?

- ☐ Lilongwe
- ☐ Dedza
- ☐ Ntchisi
- ☐ Dowa
- ☐ Kasungu
- ☐ Nkhotakota
- ☐ Ntcheu
- ☐ Mchinji
- ☐ Salima
- ☐ others (specify)

specify other

____

Which multiplier do you buy your Australorp chickens or eggs from?

- ☐ others (specify)

specify other

____

How are your black Australorp chickens kept?
mumaweta bwanji nkhuku zanu za mikolongwe

- ◯ housed all the time
- ◯ housed with access to outdoors (fenced enclosure)
- ◯ housed at night only (free ranging)

What is your purpose in keeping black Australorp chickens? (Tick all applicable)
chifukwa chani mukuweta nkhuku za mikolongwe? (chongani zonse zomwe ayankha)

- ☐ egg production (subsistence)
- ☐ meat production (subsistence)
- ☐ breeding
- ☐ egg production (commercial)
- ☐ meat production (commercial)

Do you use commercial pre-mixed feed?
mumagula chakudya cha nkhuku?

- ◯ yes
- ◯ no

» Chicken waste

What do you do with the chicken waste from the chickens? (Tick all that apply)
zitosi zankhuku zanu mumagwiritsa ntchito yanji? (chongani zonse zoyankhidwa)

- ☐ fertilizer (own use)
- ☐ sell
- ☐ biogas
- ☐ leave it where it dropped
- ☐ fish feed
- ☐ dairy feed
- ☐ other (specify)

specify other

____

Have you ever heard of a type of medicine called an antibiotic?
Munamvapo za mankhwala atchedwa antibiotic?

- ◯ yes
- ◯ no

Which of these do you consider to be an antibiotic for animal use? (VISUAL AID 1)
Pa mankhwala awa, ndi mankhwala ati amene mumawatenga kuti ndima antibiotic?

- [ ] A01
- [ ] A02
- [ ] A03
- [ ] A04
- [ ] A05
- [ ] A06
- [ ] A07
- [ ] A08
- [ ] A09
- [ ] A10
- [ ] A11
- [ ] A12

These are examples of antibiotics. Have you ever heard or used any of these? (VISUAL AID 2)

- ( ) yes
- ( ) no

Do you ever use antibiotics on the farm to treat your chickens?
Kodi mumagwiritsa ntchito mankhwala a antibiotic pa khola lanu?

- ( ) yes
- ( ) no

» Antibiotic use in animals

» » Reasons for using antibiotics

What are your main reasons for using antibiotics in your chickens? (Tick all that apply)
Ndi zifukwa ziti zomwe mumagwiritsira ntchito ma antibiotic?(chongani zonse zomwe ayankha)

- [ ] growth
- [ ] treatment
- [ ] disease prevention
- [ ] other (specify)

specify other

» » Accessing antibiotics

How do you access antibiotics for your chicken? (Tick all that apply)
Mankhwala a antibiotic mumawapeza bwanji? ?(chongani zonse zomwe ayankha)

- [ ] veterinary drug shops
- [ ] direct importation
- [ ] government offices
- [ ] friends
- [ ] other (specify)

specify other

» » Types of antibiotics used on the farm

Which types of antibiotics are commonly used on your chickens? (Tick all that apply) (VISUAL AID 2)
Ndi ma antibiotic ati amene mumagwiritsa ntchito pafupafupi pa khola panu?. (chongani zonse zimene ayankhe)

- [ ] AU-01
- [ ] AU-02
- [ ] AU-03
- [ ] AU-04
- [ ] AU-05
- [ ] AU-06
- [ ] AU-07
- [ ] AU-08
- [ ] AU-09
- [ ] AU-10
- [ ] AU-11
- [ ] AU-12
- [ ] AU-13
- [ ] AU-14
- [ ] AU-15
- [ ] other (specify)

specify other

» » Perscription practices

For the antibiotics you use, do you usually get a prescription ?
Kodi mumapeza/mumapasidwa upangiri wa kagwiritsidwe ntchito ka ma nkhwala a antibiotic kapena ayi?

- ( ) yes
- ( ) no

Who prescribes the antibiotics used for your chickens? (Tick all that apply)
Ngati mumagwiritsa ntchito upangiri wa magwiritsidwe ntchito a ma antibiotic, amakupatsani ndani upangiriwo?(chongani zonse zomwe ayankhe)

- [ ] Vet officer
- [ ] AVO
- [ ] community animal health worker
- [ ] vet shop sellers
- [ ] Other (Specify)

specify other

» Consult a vet

Do you normally consult a veterinarian when your chickens are sick?
Kodi mumafunsa mlangizi wa ziweto nkhuku zanu zikadwala nthawi zonse?

- ( ) Yes, always
- ( ) Yes, sometimes
- ( ) No

What are the reasons for not consulting a vet? (Tick all that apply)
Ngati ndi ayi zifukwa zake ndi ziti? (chongani zonse zomwe ayankhe)

- [ ] charges are exorbitant
- [ ] their services do not help
- [ ] they never show up when called
- [ ] no veterinarian available
- [ ] other (specify)

specify other

How do you administer the antibiotics to your chickens? (Tick all that apply)
Mumapereka bwanji mankhwala a antibiotic ku nkhuku zanu? (chongani zonse zomwe ayankhe)

- [ ] water
- [ ] feed
- [ ] injection
- [ ] other (specify)

specify other

For how long do you administer antibiotics to your chickens ?
nkhuku zanu mumapereka mankhwawla kwa nthawi yaitali bwanji

- ○ I stop once the chickens get better even if I don't finish administering the medicine
- ○ I finish administering medicine as directed
- ○ I buy some more medicine if the chickens don't improve
- ○ other

specify other

Have there been situations where the chickens were given antibiotics which were prescribed for human use?
Munakhalako ndi nyengo zina zomwe nkhuku zanu munazipatsa ma antibiotic a anthu?

- ○ yes
- ○ no

Which human-prescribed antibiotic(s) have you used before (VISUAL AID 3)?
ngati eya, ndi mankhwala ati amene munagwiritsako ntchito?

- [ ] H01
- [ ] H02
- [ ] H03
- [ ] H04
- [ ] H05
- [ ] H06
- [ ] H07
- [ ] H08
- [ ] H09
- [ ] H10
- [ ] other (specify)

specify other

Do you ever sell/consume chickens during treatment?
Munadyako/kugulitsa nkhuku munthawi yomwe nkhuku zanu zikulandila mankwala?

- ◯ yes
- ◯ no

How long do you usually wait after treatment ends, before you start selling/consuming your chickens?
ngati ayi, mumadikila kwa nthawi yaitali bwanji mukamaliza kupereka mankhwala musanayambe kugulitsa kapena kudya nkhuku zanu ?

- ◯ less than 5
- ◯ 5-7 days
- ◯ >7
- ◯ other

specify other

Do you ever sell/consume eggs during treatment?
Munadyako/kugulitsa mazila munthawi yomwe nkhuku zanu zikulandila mankwala?

- ◯ yes
- ◯ no

How long do you usually wait after treatment ends, before you start selling/consuming your egg?
ngati ayi, mumadikila kwa nthawi yaitali bwanji mukamaliza kupereka mankhwala musanayambe kugulitsa kapena kudya mazira anu ?

- ◯ less than 5
- ◯ 5-7 days
- ◯ >7
- ◯ other

specify other

Which of these do you consider to be antibiotics for human use (VISUAL AID 4)?
mwa zinthu izi, ndi mankhwala ati amene mumawa Tchula kuti ndi a antibiotic?

- ☐ HK01
- ☐ HK02
- ☐ HK03
- ☐ HK04
- ☐ HK05
- ☐ HK06
- ☐ HK07
- ☐ HK08
- ☐ HK09
- ☐ HK10

Have you ever heard about antibiotic resistance?
munamvako za kupima/kufuwa kwa mankhwala a antibiotic?

- ◯ yes
- ◯ no

» Resistance Source

Where did you first hear about it? (Tick all that apply)
ngati eya, ndiliti lomwe muyamba kumva zimenzi ? (chongani zonse zomwe ayankhe)

- [ ] Radio
- [ ] newspaper
- [ ] internet
- [ ] Extension worker
- [ ] other (specify)

specify other

» Awareness of AMR

Are you aware of any actions that may promote antibiotic resistance
kodi mukudziwako mchitidwe uliwonse umene ungathe kulimbikitsa kupima/kufuwa kwa mankhwala a antibiotic?

- ○ yes
- ○ no

What may promote antibiotic resistance. (Tick all that apply)
ngati eya, ndichani chingalimbikitse kupima/kufuwa kwa mankhwala a antibiotic?. (chongani zonse zomwe ayankhe)

- [ ] using antibiotics without a prescription
- [ ] not completing a prescription as directed by a health worker
- [ ] using antibiotics to treat flu
- [ ] taking antibiotics as directed by a health worker
- [ ] Buying antibiotics from the market

specify other

How would you classify the importance of antibiotic resistance? (Give them the options)
kodi mukuwona kuti nkhani ya kupima/kufuwa kwamankhwala ndiyofunikila motani

very important — important — not sure — not important — very unimportant

How strongly would you agree or disagree with the statement "antibiotic resistance is a problem for foods"
Kodi kupima/kufuwa kwa mankhwala a antibiotic mungakutenge ngati vuto ku ziweto?

strongly agree — agree — neutral — disagree — strongly disagree

How strongly would you agree or disagree with the statement "antibiotic resistance is a problem for humans?"
Kodi kupima/kufuwa kwa mankhwala a antibiotic mungakutenge ngati vuto kwa anthu?

strongly agree — agree — neutral — disagree — strongly disagree

» How you access treatment

How do you access antibiotics when you/ family members are sick? (Tick all that apply)
mumapeza bwanji ma antibiotics pamene inuyo kapena aliyense wapabanja panu wadwala? (chongani zonse zomwe ayankha)

- [ ] government offices/CHAM facility
- [ ] private clinic
- [ ] pharmacy (prescription)
- [ ] pharmacy (without a prescription)
- [ ] market
- [ ] Use left-over antibiotics
- [ ] other (specify)

specify other

» Use without a perscription

Do you ever use antibiotics for you or your family without a prescription?
munagwiritsako ntchito mankhwala antibiotic popanda uphungu wa ogwira ntchito kuchipatala?

- ( ) yes
- ( ) no

Why do you sometimes not get a prescription from a health worker? (Tick all that are applicable)
ngati eya, ndichifukwa chiyani simupeza uphungu kuchokera kwa anthu ogwira ntchito kuchipatala? (chongani zonse zimene ayankha)

- [ ] Charges are exorbitant
- [ ] Already knows which medication to buy
- [ ] Medicines can be accessed without prescription
- [ ] The health facility is too far
- [ ] Other (Specify)

specify other

» What conditions do you use antibiotics in

Have you taken antibiotics for these conditions? (Tick all that apply)
munagwiritsako ntchito mankhwala a antibiotic pamene munali ndi mavuto awa? (chongani zonse zomwe anena)

- [ ] Chimfine
- [ ] Chimfine chopwanyisa thupi
- [ ] covid
- [ ] pneumonia
- [ ] cough
- [ ] sore throat
- [ ] fever
- [ ] diarrhoea
- [ ] other (specify)

specify other

» length of time antibiotics taken

Which option best describes how long you take a course of antibiotics for? (Read out options)
mumagwiritsa ntchito ma antibiotic kwanthawi yaitali motani?

- ◯ I stop once I feel better even if I don't finish the course
- ◯ Finish the course as directed by the doctor
- ◯ I buy some more antibiotics if I don't improve
- ◯ others (specify)

specify other

» Now collect samples

1

Select specimen type

- ◯ human
- ◯ chicken
- ◯ environment

Is this sample from the person answering the questionnaire

- ◯ yes
- ◯ no
- ◯ don't know

» » barcode scanning

Scan sample barcode

Did scan fail (if yes manually enter below)

- ◯ yes
- ◯ no

* **Manually enter sample code (first time)**

---

* **Manually enter sample code (second)**

Thank you for your time
Zikomo chifukwa cha nthawi yanu
